# Effects and interaction of meteorological factors on hemorrhagic fever with renal syndrome incidence in Huludao City, northeastern China, 2007–2018

**DOI:** 10.1371/journal.pntd.0009217

**Published:** 2021-03-25

**Authors:** Wanwan Sun, Xiaobo Liu, Wen Li, Zhiyuan Mao, Jimin Sun, Liang Lu

**Affiliations:** 1 Zhejiang Provincial Center for Disease Control and Prevention, Hangzhou, China; 2 State Key Laboratory of Infectious Disease Prevention and Control, Collaborative Innovation Center for Diagnosis and Treatment of Infectious Disease, National Institute for Communicable Disease Control and Prevention, Chinese Center for Disease Control and Prevention, Beijing, China; 3 Cornell University, Ithaca, New York, United States of America; Chengde Medical University, CHINA

## Abstract

**Background:**

Hemorrhagic fever with renal syndrome (HFRS), a rodent-borne disease, is a severe public health threat. Previous studies have discovered the influence of meteorological factors on HFRS incidence, while few studies have concentrated on the stratified analysis of delayed effects and interaction effects of meteorological factors on HFRS.

**Objective:**

Huludao City is a representative area in north China that suffers from HFRS with primary transmission by *Rattus norvegicus*. This study aimed to evaluate the climate factors of lag, interaction, and stratified effects of meteorological factors on HFRS incidence in Huludao City.

**Methods:**

Our researchers collected meteorological data and epidemiological data of HFRS cases in Huludao City during 2007–2018. First, a distributed lag nonlinear model (DLNM) for a maximum lag of 16 weeks was developed to assess the respective lag effect of temperature, precipitation, and humidity on HFRS incidence. We then constructed a generalized additive model (GAM) to explore the interaction effect between temperature and the other two meteorological factors on HFRS incidence and the stratified effect of meteorological factors.

**Results:**

During the study period, 2751 cases of HFRS were reported in Huludao City. The incidence of HFRS showed a seasonal trend and peak times from February to May. Using the median WAT, median WTP, and median WARH as the reference, the results of DLNM showed that extremely high temperature (97.5^th^ percentile of WAT) had significant associations with HFRS at lag week 15 (RR = 1.68, 95% CI: 1.04–2.74) and lag week 16 (RR = 2.80, 95% CI: 1.31–5.95). Under the extremely low temperature (2.5^th^ percentile of WAT), the RRs of HFRS infection were significant at lag week 5 (RR = 1.28, 95% CI: 1.01–1.67) and lag 6 weeks (RR = 1.24, 95% CI: 1.01–1.57). The RRs of relative humidity were statistically significant at lag week 10 (RR = 1.19, 95% CI: 1.00–1.43) and lag week 11 (RR = 1.24, 95% CI: 1.02–1.50) under extremely high relative humidity (97.5^th^ percentile of WARH); however, no statistically significance was observed under extremely low relative humidity (2.5^th^ percentile of WARH). The RRs were significantly high when WAT was -10 degrees Celsius (RR = 1.34, 95% CI: 1.02–1.76), -9 degrees Celsius (1.37, 95% CI: 1.04–1.79), and -8 degrees Celsius (RR = 1.34, 95% CI: 1.03–1.75) at lag week 5 and more than 23 degrees Celsius after 15 weeks. Interaction and stratified analyses showed that the risk of HFRS infection reached its highest when both temperature and precipitation were at a high level.

**Conclusions:**

Our study indicates that meteorological factors, including temperature and humidity, have delayed effects on the occurrence of HFRS in the study area, and the effect of temperature can be modified by humidity and precipitation. Public health professionals should pay more attention to HFRS control when the weather conditions of high temperature with more substantial precipitation and 15 weeks after the temperature is higher than 23 degrees Celsius.

## Author summary

Climate change impacts vector-borne disease incidence by influencing vectors’ habitat and behaviors. As a rodent-borne disease, HFRS’s incidence rate fluctuates with the change of meteorological factors. In this study, we model the meteorological factors and time-series cases to explore the exposure-lag-response effect and interaction between meteorological factors on the risk of HFRS, respectively. The result showed there exist a lag effect between meteorological factors and the occurrence of HFRS and we find that a temperature higher than 23 Celsius degrees resulted in a significantly higher HFRS incidence after 15 weeks; a relative humidity higher than 93% led to a significantly higher incidence after 10 weeks. Also, a synergistic interaction between high temperature and high precipitation on HFRS risk was detected, this effect can be attributed to increased animal reproduction and food resources under this environment. This study provides a basis for in-depth evaluating the impact of meteorological factors and their interaction on HFRS.

## Introduction

Hemorrhagic fever with renal syndrome (HFRS) characterized by headache, fever, back pain, abdominal pain, and acute renal dysfunction has caused a series of public issues, with 30,000–60,000 cases annually in the 1990s in mainland China[1,2]^.^ China has the highest HFRS incidence globally, in which domestic HFRS cases account for 90% of total cases worldwide each year[3,4]. Although the number of HFRS cases decreased substantially from more than 1 million cases between 1950 and 1995 to 10 000 cases annually in 2009, the geographical distribution of HFRS cases has further expanded, and HFRS has been reported in all 31 provinces in mainland China[5].

Hantaan virus (HTNV) and Seoul virus (SEOV) are the predominantly causative pathogens of HFRS, with HTNV-caused cases accounting for approximately 70% of total domestic HFRS case[6]. Both HTNV and SEOV strains have spread across China, and the two virus strains can exist in rodents in the same area or different areas separately, leading to spring outbreaks or autumn outbreaks in turn[7]. The main natural reservoir hosts of HTNV and SEOV are the striped field mouse (*Apodemus agrarius*) and the brown Norway rat (*Rattus norvegicus*), respectively[8]. Although HFRS can occur across a year, there is generally a bimodal seasonal distribution with a rapid peak in spring and a long-lasting peak in autumn to winter[9,10]. Generally, the autumn to winter peak is mainly associated with *A*. *agrarius*, which is abundant in farmland regions, while the spring epidemic peak is mainly associated with *R*.*s norvegicus* common in urban areas.

Climatic condition is widely considered one of the most critical factors that can affect rodent population dynamics and lead to more HFRS cases in humans as a consequence[11]. Previous studies have researched the associations between climatic factors and HFRS epidemic risks[12–14]. Zhang et al. found that monthly average air temperature was nonlinearly correlated with the monthly incidence of HFRS and reached the highest relative risk (RR) at approximately 23 degrees Celsius in Shandong Province[15]. A study carried out in Guangzhou indicated that lags in temperature from 1–3 months, rainfall of 2 months and relative humidity of 4 months all have significant associations with the incidence of HFRS. Correlation analysis was performed using the Pearson correlation method[16]. Evidence indicates that humidity, temperature and precipitation may affect the incidence of HFRS[17]. Since multiple meteorological factors exist at the same time and constitute a specific climatic condition, we explored the relationship between meteorological factors and HFRS and believed that the interaction among meteorological factors on the occurrence of HFRS is also worthy of attention. Few studies have comprehensively researched the lag effect of multiple meteorological factors on the occurrence of HFRS and conducted interaction analyses among them.

DLNM represents a modeling framework to simultaneously describe nonlinear and delayed dependencies and thus explore exposure-lag-response associations[18]. GAM is the extension of linear regression analysis; it allows for adjustments of the nonparametric, nonlinear, and confounding effects of seasonality, which have been previously used in modeling time-scale data and to explore the interaction effects between variables[19]. Our study used DLNM and GAM to explore the lag and interaction effects of temperature, precipitation, and humidity using infectious disease surveillance data of HFRS in Huludao City. Our results provide more evidence to support decision-making for the prevention and control of HFRS under different weather conditions.

## Methods

### Setting

**Fig 1:**
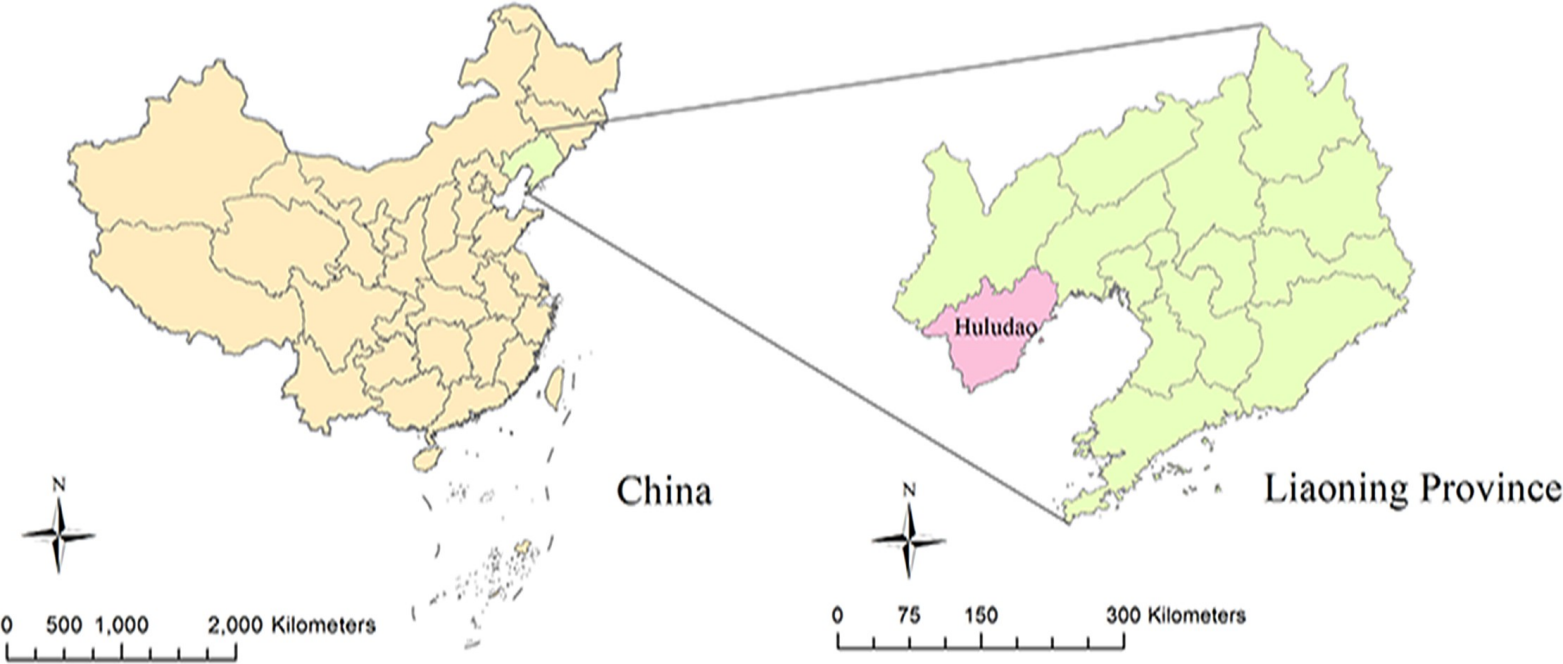
The geographical location of Huludao City in China. The map was created by ArcGIS 10.3 (Environmental Systems Research Institute; Redlands, CA, USA). The base map was acquired from the data center for geographic sciences and natural sources research, CAS (http://www.resdc.cn/data.aspx?DATAID=201).

Huludao City, a coastal city of Liaoning Province of China, is located between longitudes 119°12′ and 121°02′ E and between latitudes 39°59′ and 40°56′ N. In 2018, Huludao City’s total population was 2.76 million. Huludao City belongs to the north temperate climate zone and has an annual average relative humidity of 64.8%, a weekly average temperature of 9.48 degrees Celsius, and annual precipitation of 534 mm (Fig 1).

### Data collection

We obtained the surveillance data for this study from HFRS cases in Huludao City between 2007 and 2018 from the National Center for Disease Control and Prevention of China. All patients were diagnosed based on the criteria and principles of management for HFRS issued by the Ministry of Health of the People’s Republic of China.

Meteorological data over the same period, weekly average temperature (WAT, degrees Celsius), weekly average relative humidity (WARH, percentage), and weekly total precipitation (WTP, millimeters) were calculated from the corresponding daily data obtained from the China Meteorological Data Sharing Service System (www.data.cma.cn), which is accessible for free. Original data of HFRS and meteorological can be found in S1 Data.

### Data analysis

A descriptive analysis was first performed to describe the temporal trend of HFRS cases and meteorological factors during the study period in Huludao City. The meteorological factors were chosen based on the findings of previous studies on the association between climatic factors and HFRS, including temperature, humidity, and precipitation. Then, DLNMs were created to estimate the delayed effect of meteorological factors on HFRS incidence[14,20,21]. The lag phase between meteorological change and the change of the HFRS epidemic, most commonly several weeks is length, consists of bacterium transmission to humans from other host species and the incubation period in human bodies[21–23]. Typically, in our study, the maximum lag period was set as 16 weeks because Hardestam et al. found that HTNV can survive outside of hosts under wet conditions for as long as 96 days[24], and its incubation periods in the human body range from 1 to 4 weeks (most common in 7–14 days)[25]. As weekly HFRS new infections in Huludao City are rare compared to the city’s population, and quasi-Poisson were used in the models of this study to control overdispersion.

To analyze the lag-response effect of meteorological factors, WAT, WTP, and WARH were applied to the cross-basis function of DLMN. When one factor was included in the function, the other two were set as covariate variables (see the model below). Relative risk (RR) was calculated to evaluate the effect of meteorological factors with different lag weeks on HFRS incidence.

The model can be written as follows:

$$\log\left[E(Yt)\right]=\beta +cb(K_{t,16},\beta 1)+s_{1}(X)+s_{2}(Z)+s_{3}(week)$$

Here, *Yt* was the number of weekly counts of HFRS cases in week t; *β* was the intercept of the whole model; *cb*(*K*_*t*,16_,*β*1) was the cross-basis function for K which was one of the meteorological factors (WAT, WTP, and WARH), and X, Z denotes the other two; *β*1 was the effect estimate of K at the specific lag week t; 16 was the largest lag week in this study; “Week” was the date of different observations; s() indicates penalized spline function. The spline functions, denoted as s_1_(X) and s_2_(Z), were used to adjust for confounders, and s_3_(week) was used to adjust for weekly confounding in the model. The optimal degrees of freedom (df) for the spline function were estimated by generalized cross-validation (GCV) criteria[26,27].

Second, GAMs were conducted to explore the interaction and stratification effects of meteorological factors on the HFRS epidemic.

The model can be written as follows:

$$\log\left[E(Yt)\right]=\beta 2+s_{1}(K,X)+s_{2}(Z)+s_{3}(week)$$

*β*2 is the intercept; K denotes one of the meteorological factors (WAT, WTP, and WARH), and X and Z denote the other two. s() indicates penalized spline function. s_1_ (K, X) is the spline function of the interaction between variables K and X. The interaction effect between WAT and WTP was explored first, followed by WAT and WARH.

Then, to explore the stratified effect of modification and qualitatively study the association between temperature and HFRS incidence by humidity and precipitation, we divide WARH and WTP into two categories—“low” and “high”—by their median value. Within this model, WAT was entered as the continuous variable. Here, the spline function of s_1_(K,X) used to analyze the interaction between two variables was deleted, (K,X) was directly put into the model for calculation, and s_2_(Z) and s_3_(week) were the spline functions to adjust the confounders.

All analyses in our study were performed using the “dlnm”, “mgcv” and “pheatmap” packages in R software (version 3.6.1). The test results of the variables in the GAMs and DLNMs are listed in S1 Text. The confidence interval of all two-sided statistical tests in the study was set as 95%, and P<0.05 was considered statistically significant.

## Results

### Descriptive analysis

**Fig 2:**
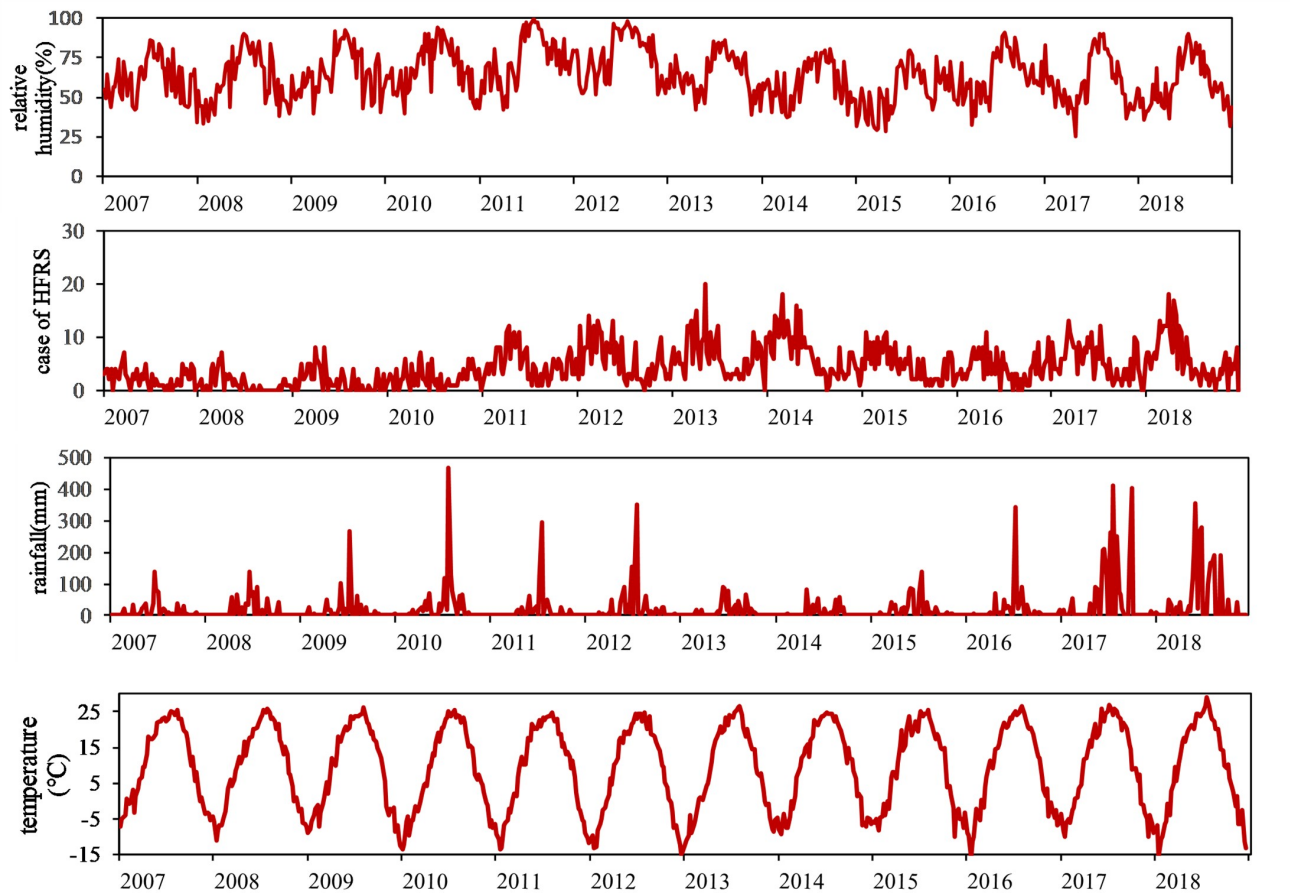
Time series plot of the HFRS cases and meteorological factors.

**Fig 3:**
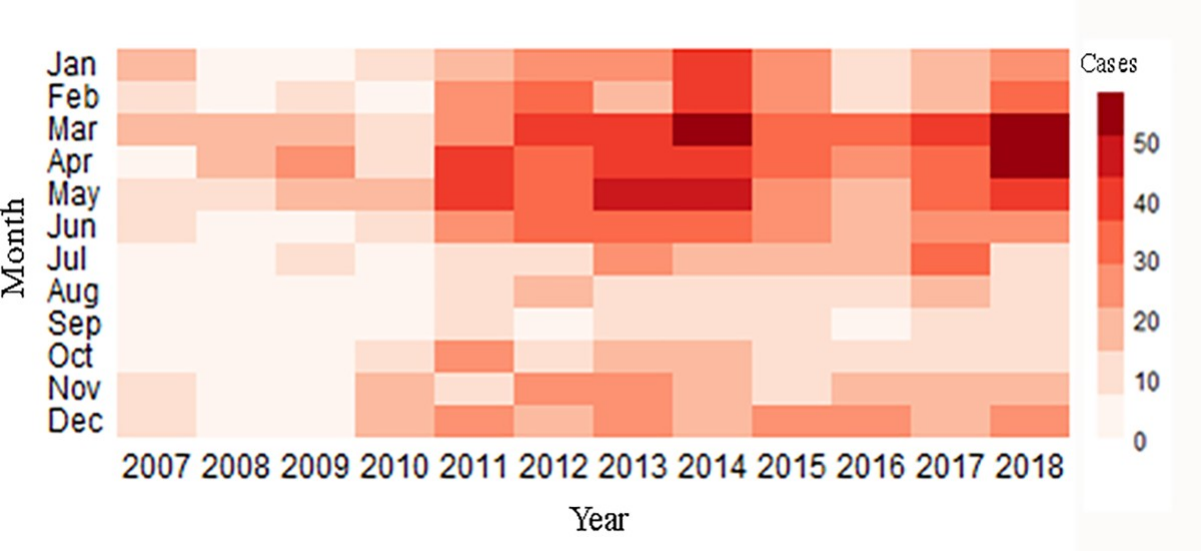
Monthly distribution of HFRS cases.

A total of 2751 HFRS cases were reported in Huludao City in the study period. The summary statistics of all the HFRS cases and meteorological variables of Huludao City are shown in Table 1. The weekly average values of HFRS case number, temperature, and relative humidity were 4.33, 9.24 degrees Celsius, and 64%, respectively. The weekly total precipitation was 20.84±54.00 mm. The temporal trends of HFRS cases and meteorological factors are shown in Fig 2, which suggests a potential association between climate factors and HFRS incidence and a seasonal HFRS case distribution pattern. The heatmap in Fig 3 correspondingly describes a clear seasonal pattern of HFRS case distribution, with most cases reported in spring (February to May), which accounted for 48.71% of the total cases each year.

**Table 1:** Summary statistics of weekly HFRS cases and meteorological factors in Huludao City, China, 2007–2018.

Variable	Mean	S.D.	Min	25th	Median	75th	Max
Case of HFRS	4.33	3.57	0.00	2.00	3.00	6.00	20.00
Mean temperature (celsius degree)	9.24	11.92	-16.20	-2.11	10.53	20.38	28.96
Relative humidity (%)	63.89	15.53	25.57	52.14	63.00	75.50	99.29
Precipitation (mm)	20.84	54.00	0.00	0.00	0.43	17.36	466.71

Abbreviations: HFRS, Hemorrhagic fever with renal syndrome; S.D., the standard deviation; Min, the minimum of variables; Max, the maximum of variables. All the data are presented as weekly average or aggregate values.

### The lag relationship between meteorological factors and the incidence of HFRS

**Fig 4:**
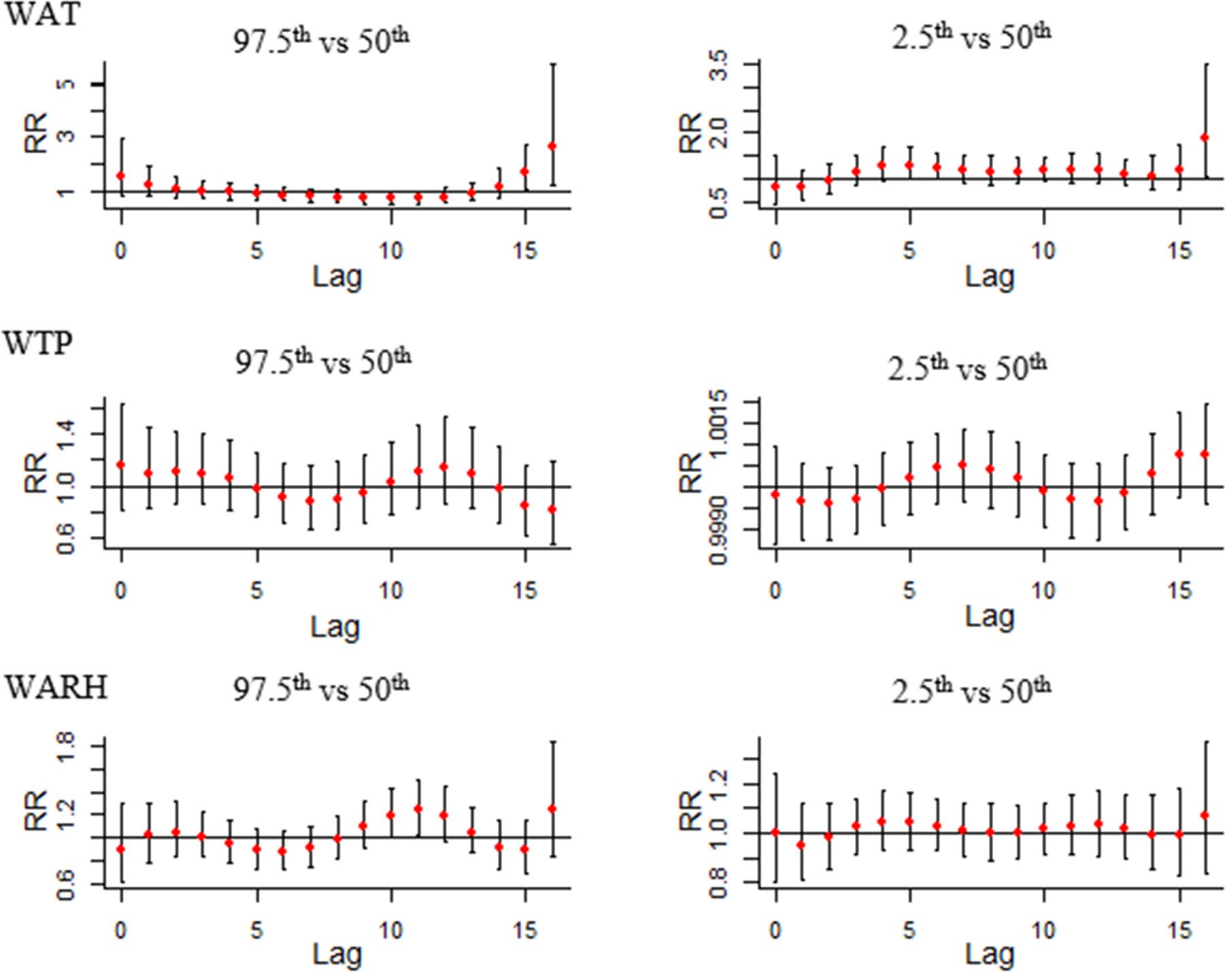
The lag effect between WAT, WTP, WARH, and HFRS infection (Abbreviations: RR, relative risk; WAT, weekly average temperature; WTP, weekly total precipitation; WARH, weekly average relative humidity).

The results of DLNMs are shown in Fig 4. In DLNMs, we set median WAT, median WTP, and median WARH as the reference, and then the RR with 95% confidence intervals of HFRS infection among lag weeks was calculated with the 97.5^th^ and 2.5^th^ percentile of WAT, WTP, and WARH, respectively. The slice plot presented in Fig 4 shows that under the extremely high temperature (97.5^th^ percentile of WAT), the RRs for lag week 15 (RR = 1.68, 95% CI: 1.04–2.74) and 16 weeks (RR = 2.80, 95% CI: 1.31–5.95) are significantly high. Furthermore, under extremely low temperature (2.5^th^ percentile of WAT), the RRs of HFRS infection were significantly high for a lag of 5 weeks (RR = 1.28, 95% CI: 1.01–1.67) and 6 weeks (RR = 1.24, 95% CI: 1.01–1.57). However, in the WTP slice plots, no statistical significance in RRs was observed when comparing extremely high total precipitation (97.5^th^ percentile of WTP) with median precipitation and between extremely low total precipitation (2.5^th^ percentile of WTP) and median precipitation. In WARH slice plots, the RRs were significantly high for a lag of 10 weeks (RR = 1.19, 95% CI: 1.00–1.43) and 11 weeks (RR = 1.24, 95% CI: 1.02–1.50) under extremely high relative humidity (97.5^th^ percentile of WARH); however, no statistically significant difference was observed under extremely low relative humidity (2.5^th^ percentile of WARH).

**Fig 5:**
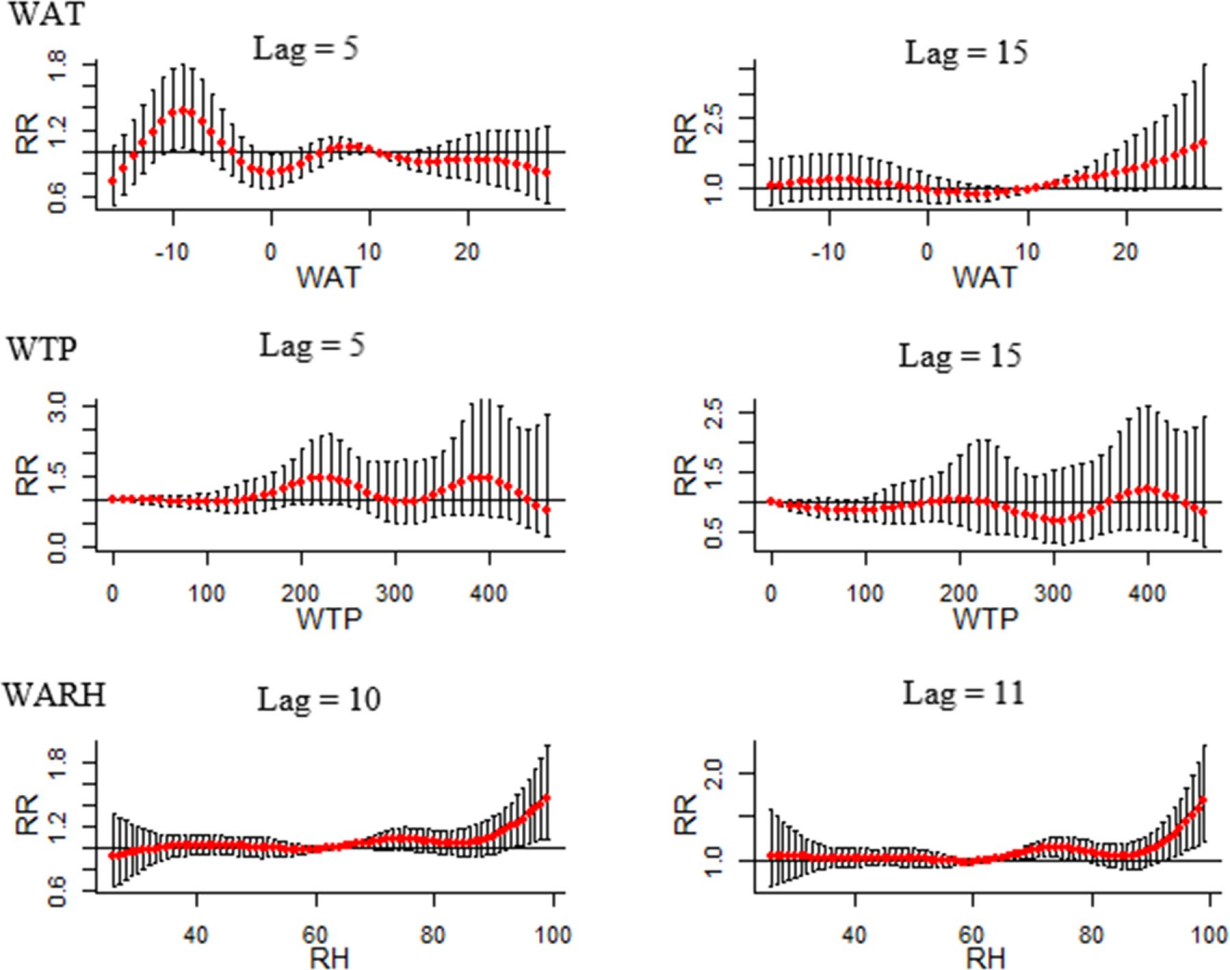
The lag-specific effect of climate factors on HFRS (Abbreviations: RR, relative risk; WAT, weekly average temperature; WTP, weekly total precipitation; WARH, weekly average relative humidity).

Fig 5 shows the lag-specific association between different meteorological factors and HFRS incidence. WAT slice plots show that RRs were significantly high when WAT was -10 degrees Celsius (RR = 1.34, 95% CI: 1.02–1.76), -9 degrees Celsius (1.37, 95% CI: 1.04–1.79) and -8 degrees Celsius (RR = 1.34, 95% CI: 1.03–1.75) after 5 weeks. A temperature higher than 23 degrees Celsius resulted in a significantly higher RR value after 15 weeks. In WARH slice plots, significant RRs were observed when WARH exceeded 93% at both lags of 10 and 11 weeks. No significant RR was observed in WTP slice plots.

### Meteorological interaction and stratified analysis during WAT, WARH, WTP and HFRS cases

**Fig 6:**
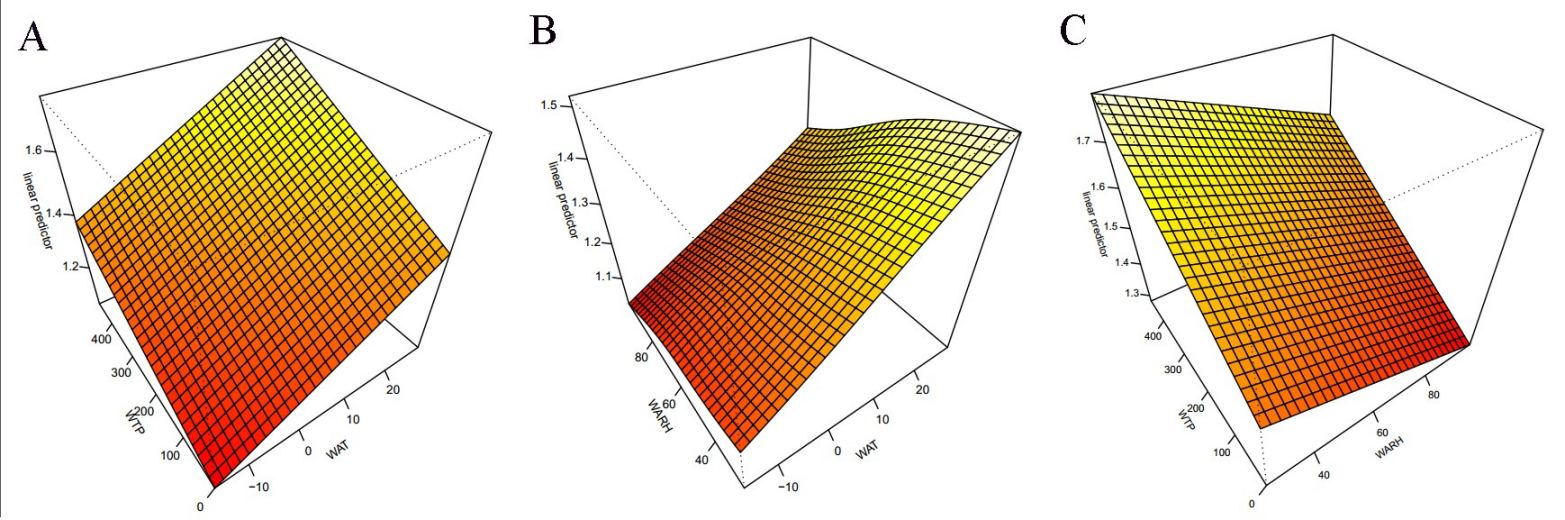
The effect interactions of the association among temperature, relative humidity, precipitation and HFRS in Huludao, 2007–2018. (Abbreviations: RR, relative risk; WAT, weekly average temperature; WTP, weekly total precipitation; WARH, weekly average relative humidity. A: the interaction effect between WAT and WTP; B: the interaction effect between WAT and WARH; C: the interaction effect between WARH and WTP).

Statistically significant GAMs were constructed to show the interaction effect among WAT, WARH, and WTP on HFRS incidence (Fig 6). The picture on the left side of Fig 6 suggests the interaction effect of temperature and precipitation on HFRS. The HFRS infection risk increased as weekly average temperature and precipitation increased. The picture in the middle of Fig 6 shows the interaction effect of temperature and relative humidity on HFRS. The risk of HFRS infection increased with increasing weekly average temperature and decreasing relative humidity. The picture to the right of Fig 6 suggests the interaction effect of relative humidity and precipitation, HFRS tends to occur in higher precipitation and lower relative humidity weather conditions. The risk of HFRS infection was highest when both temperature and precipitation were at the highest level.

**Fig 7:**
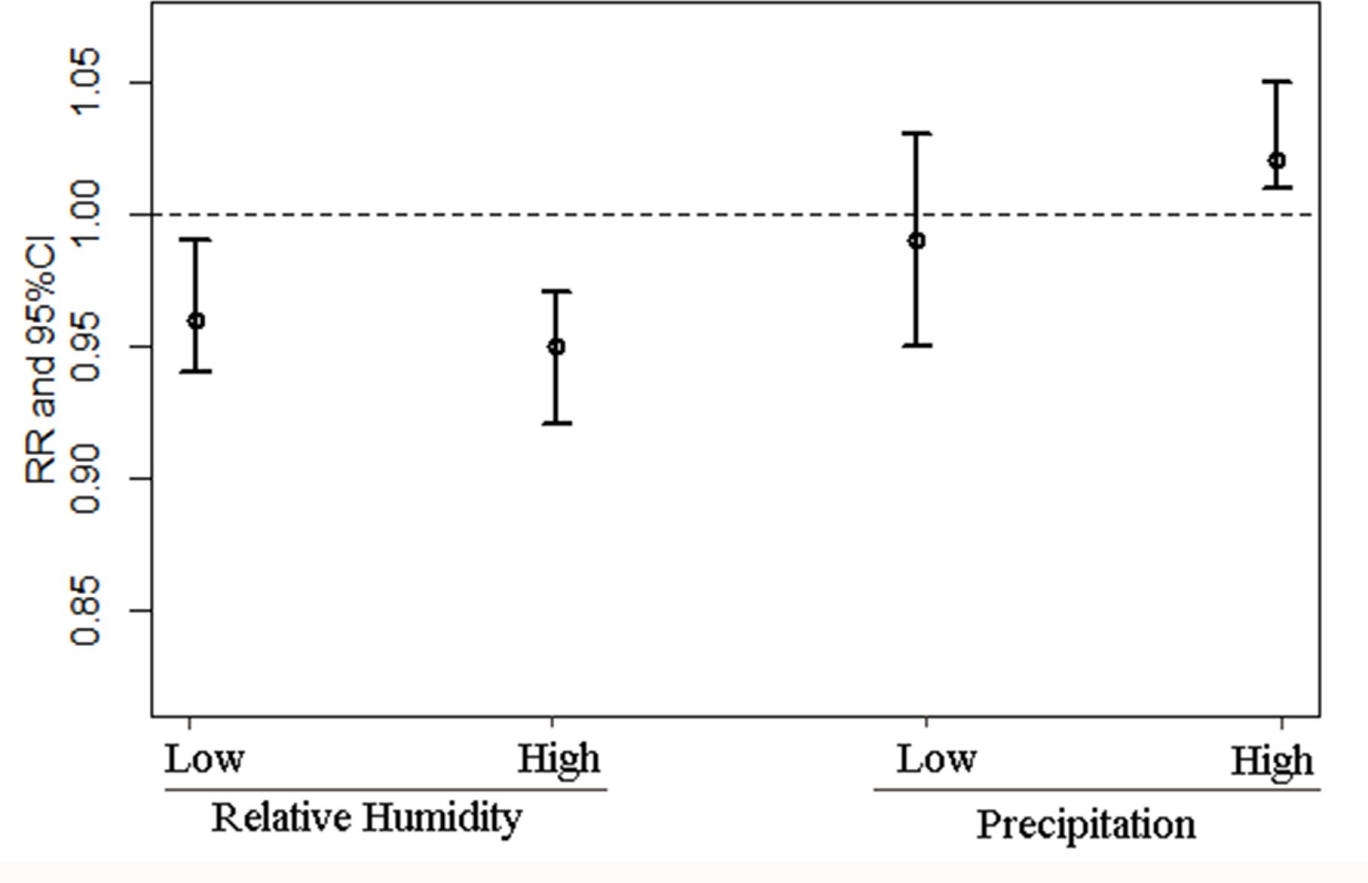
The associations between temperature and HFRS with different strata of relative humidity and precipitation (Abbreviations: RR, relative risk; CI: confidence interval).

The stratified analysis (Fig 7) showed that relative humidity and precipitation could modify the association between temperature change and HFRS infection risk. In a low relative humidity environment, a 1 degree Celsius increase will lead to a 4% (95% CI: 1%-6%) decrease in HFRS infection risk, while in a high relative humidity environment, a 1 degree Celsius increase will lead to a 5% (95% CI: 3%-8%) decrease in HFRS infection risk. In a high precipitation environment, a 1 degree Celsius increase will lead to a 2% (95% CI: 1%-5%) increase in HFRS infection. In comparison, no significant change was observed in a low precipitation environment.

## Discussion

In this study, the delay, interaction and stratified effects of meteorological factors on the HFRS epidemic in Huludao City were explored. Our study results show that temperature and humidity have delayed effects on the occurrence of HFRS. In addition, our study also suggests that rising temperatures and increasing precipitation can collectively boost the risk of HFRS infection.

We used a DLNM to estimate the weekly lag effect of climatic factors on the occurrence of HFRS and found that the lag effects of different climatic factors were not all the same. The different lag periods reflected that the delayed effect of each climatic variable may be associated with the transmission of the infection being affected by various factors, including the proliferation of the virus in the external environment, people’s tendency to go out, seasonal variation in the rodent population and so on[28,29]. Joshi et al. studied the influence of climatic factors on the development of HFRS during the peak season and found that temperature at a lag of 11 weeks had the largest RR[30]. Seasonal autoregressive integrated moving average models (SARIMAX) conducted by He et al. in two countries in Northeast China showed that rainfall with a 3–4 month lag was closely correlated with HFRS, whereas relative humidity with a 1–5 month lag significantly impacted HFRS transmission[31]. The highest temperature in a year occurs between June and September, while the occurrence number of HFRS cases peaks between September and December, suggesting that HFRS incidence may lag behind the temperature by approximately 3 months[32].

HFRS is a zoonotic disease, with most cases identified in the spring season in most cities (such as Huludao City and Guangzhou). However, other than in the spring season, HFRS cases are also reported at a high frequency during the winter season (November to January) in Shenyang, the capital city of Liaoning Province, which is 280 km away from Huludao City[33]. Compared with Huludao City, Shenyang City has a lower annual mean temperature and less precipitation[10]. Other than the common rodent species of *R*. *norvegicus* endemic in Huludao City, *A*. *agrarius* was also endemic in Shenyang City and caused two epidemic peaks each year[16].

Consistent with previous studies, our study found that rising temperatures increased the risk of HFRS infection[13,34]; for example, Liu et al. found that temperatures between 10–25 degrees Celsius was a favorable condition for HFRS transmission in Junan County in Shandong[14]. Xiang et al pooled the results of China’s 19 cities and showed that a 1°C increase in temperature resulted in a 1.6% (95% CI, 1.0%-2.2%) increase in HFRS[35]. In addition, Tian et al.’s study revealed that temperature impacts HFRS incidence in several ways, including influencing the survival rate and density of rodent species, influencing outdoor human engagement, and hence influencing the transmission of virus strains[36].

The interaction analysis results in our study indicated that higher temperatures and more substantial precipitation are risk climate conditions for the occurrence of HFRS. This result was consistent with previous studies[37]. Harvell et al. studied the impact of climate warming on vector-borne diseases and found that warmer conditions would contribute to promoting vector capacity and the basic reproductive ratio of this disease[38]. A geographic distribution analysis from Lin et al. revealed that areas with the highest incidence of HFRS were reported to have a semi-humid climate with a mountainous geographical structure in China[39]^.^

Our study of lag results did not suggest any significant association between precipitation and HFRS incidence, while we found that higher precipitation could enhance the effect of temperature on HFRS incidence based on our stratified analysis. Studies exploring the effect of rainfall on HFRS found that adequate rainfall provided a suitable survival environment and sufficient food for rodents and presented an increased risk for virus transmission[40,41]. However, rainfall would be a risk factor for HFRS infection when it exceeds a certain amount, and excessive rainfall may destroy the nests of host animals and make it difficult for them to obtain food[42,43]. While this negative effect of large precipitation was not identified in Huludao City in our study, the probable reason might be that Huludao City belongs to the temperate continental monsoon climate[44], in which the annual rainfall is 600–1200 mm, with limited flooding.

Our study’s strengths include the following: a) the study period is long, and the study collected data from multiple years. b) Our study applied advanced statistical methods, including DLNM and GAM, to analyze the delayed, interaction and stratified effects of meteorological factors and to quantitatively and qualitatively evaluate the effects of meteorological factors on HFRS incidence. Our study results can provide the first evidence of the lag effect and interaction effects of meteorological evidence on HFRS, which can guide future prevention and control strategies of HFRS.

There are several limitations to our study. First, the study did not include information on vaccination in Huludao City due to data availability. Second, although the HFRS data downloaded from the disease management system were believed to be accurate to some extent, HFRS cases remain underreported because of the problematic access to medical resources or mild symptoms and lack of help from hospitals[45]. Last, with the rapid urbanization of China, economic and population factors may also play a role in the biological transmission of HFRS.

## Supporting information

S1 Data HFRS cases and meteorological factors in Huludao City, 2007–2018. (XLSX)

S1 Text Test result of GAMs and DLNMs. (DOCX)
