# Supplementary material for: Effects and interaction of meteorological factors on hemorrhagic fever with renal syndrome incidence in Huludao City, northeastern China, 2007–2018
Source: PLoS Negl Trop Dis. 2021 Mar 25;15(3):e0009217. doi: 10.1371/journal.pntd.0009217 (PMC7993601; doi:10.1371/journal.pntd.0009217)
Supplement: S1 Text — (DOCX) [file pntd.0009217.s002.docx]

**S2 Test results of GAMs and DLNMs**

**1.Test results of DLNMs for each selected meteorological variable.**

Table 1 Distributed lag non-linear model analysis of WAT

| Variables | Edf | Ref.df | F-value | P-value |
| --- | --- | --- | --- | --- |
| WARH | 2.543 | 2.872 | 12.342 | 0.000 |
| Week | 3.784 | 4.211 | 9.670 | 0.000 |
| WTP | 4.325 | 4.563 | 10.67 | 0.000 |

Abbreviations: WAT, weekly average temperature; WTP, weekly total precipitation; WARH, weekly average relative humidity; Edf, effective degrees of freedom; Ref.df, reference degrees of freedom; F-value is the value of variables using F test.

Table 2 Distributed lag non-linear model analysis of WTP

| Variables | Edf | Ref.df | F-value | P-value |
| --- | --- | --- | --- | --- |
| WARH | 3.282 | 4.655 | 7.665 | 0.000 |
| Week | 7.942 | 8.702 | 5.263 | 0.000 |
| WAT | 4.435 | 5.443 | 6.675 | 0.000 |

Abbreviations: WAT, weekly average temperature; WTP, weekly total precipitation; WARH, weekly average relative humidity; Edf, effective degrees of freedom; Ref.df, reference degrees of freedom; F-value is the value of variables using F test.

Table 3 Distributed lag non-linear model analysis of WARH

| Variables | Edf | Ref.df | F-value | P-value |
| --- | --- | --- | --- | --- |
| WAT | 6.756 | 7.469 | 10.683 | 0.000 |
| Week | 8.309 | 8.846 | 9.696 | 0.000 |
| WTP | 7.809 | 6.970 | 8.582 | 0.000 |

Abbreviations: WAT, weekly average temperature; WTP, weekly total precipitation; WARH, weekly average relative humidity; Edf, effective degrees of freedom; Ref.df, reference degrees of freedom; F-value is the value of variables using F test.

**2.Test results of GAMs for interaction analysis.**

Table 4 Model test of the interaction analysis between WAT and WARH

| Variables | Edf | Ref.df | F-value | P-value |
| --- | --- | --- | --- | --- |
| WAT, WARH | 2.781 | 3.448 | 14.557 | 0.000 |
| Week | 7.991 | 8.730 | 19.028 | 0.000 |
| WTP | 8.332 | 7.292 | 18.242 | 0.000 |

Abbreviations: WAT, weekly average temperature; WTP, weekly total precipitation; WARH, weekly average relative humidity; Edf, effective degrees of freedom; Ref.df, reference degrees of freedom; F-value is the value of variables using F test.

Table 5 Model test of the interaction analysis between WAT and WTP

| Variables | Edf | Ref.df | F-value | P-Value |
| --- | --- | --- | --- | --- |
| WAT, WTP | 1.92 | 2.138 | 74.149 | 0.000 |
| Week | 7.363 | 10.055 | 2.90 | 0.001 |
| WTP | 8.023 | 8.743 | 20.042 | 0.000 |

Abbreviations: RR, relative risk; WAT, weekly average temperature; WTP, weekly total precipitation; WARH, weekly average relative humidity; Edf, effective degrees of freedom; Ref.df, reference degrees of freedom; F-value is the value of variables using F test.

Table 6 Model test of the interaction analysis between WARH and WTP

| Variables | Edf | Ref.df | F-value | P-Value |
| --- | --- | --- | --- | --- |
| WARH, WTP | 2.000 | 2.000 | 34.056 | 0.000 |
| Week | 8.023 | 8.743 | 20.042 | 0.000 |
| WAT | 1.950 | 2.545 | 5.743 | 0.021 |

Abbreviations: RR, relative risk; WAT, weekly average temperature; WTP, weekly total precipitation; WARH, weekly average relative humidity; Edf, effective degrees of freedom; Ref.df, reference degrees of freedom; F-value is the value of variables using F test.

**3.Test results of GAMs for stratified analysis.**

Table 7 Model test of the stratified analysis of WARH

| Variables | Estimate | Std.Error | T-value | P-value |
| --- | --- | --- | --- | --- |
| Intercept | 1.862 | 0.053 | 35.072 | 0.000 |
| WeeK | -0.935 | 0.137 | -6.836 | 0.000 |
| WTP | 0.449 | 0.377 | 1.191 | 0.234 |
| WAT: WARH (low) | 0.014 | 0.004 | 3.934 | 0.000 |
| WAT:WARH (high) | -0.016 | 0.004 | -4.317 | 0.000 |

Abbreviations: RR, relative risk; WAT, weekly average temperature; WTP, weekly total precipitation; WARH, weekly average relative humidity; Std.Error, standard error; T-value is the value of variables using T test.

Table 8 Model test of the stratified analysis of WTP

| Variables | Estimate | Std.Error | T-value | P-value |
| --- | --- | --- | --- | --- |
| Intercept | 2.583 | 0.125 | 20.545 | 0.000 |
| WeeK | -1.765 | 0.244 | -7.222 | 0.000 |
| WARH | -1.178 | 0.284 | -4.150 | 0.000 |
| WAT: WTP (low) | -0.013 | 0.010 | -1.303 | 0.194 |
| WAT: WTP (high) | -1.026 | 0.005 | -3.254 | 0.000 |

Abbreviations: RR, relative risk; WAT, weekly average temperature; WTP, weekly total precipitation; WARH, weekly average relative humidity; Std.Error, standard error; T-value is the value of variables using T test.
